# Effects of Photobiomodulation on Oral Mucositis: Visualization and Analysis of Knowledge

**DOI:** 10.3390/life12111940

**Published:** 2022-11-21

**Authors:** Wallacy Watson Pereira Melo, Walessa Alana Bragança Aragão, Daiane Claydes Baia-da-Silva, Priscila Cunha Nascimento, Rafael Rodrigues Lima, Renata Duarte de Souza-Rodrigues

**Affiliations:** Laboratory of Functional and Structural Biology, Institute of Biological Sciences, Federal University of Pará, Belém 66075-110, Brazilwalessa.aragã

**Keywords:** photobiomodulation, oral mucositis, cancer, chemotherapy, radiotherapy

## Abstract

This review article mapped and analyzed the most cited articles on the association of photobiomodulation (PBM) with oral mucositis (OM) and the evolution of clinical protocols in the area. A comprehensive search was performed on the Web of Science Core Collection (WoS-CC) database, leading to the extraction of information such as title, authors, abstract, journal name, number, average of citations, study design, year of publication, institutions, continents, countries, type of laser used, irradiated anatomical points, primary anti-cancer therapy, and laser parameters. Among those, clinical trials and literature reviews were the most common study designs. The main type of laser used was the InGaAlP diode, with a wavelength ranging from 630–660 nm, power going in 40–100 mW, and energy density ranging from 0.375–22 J/cm^2^. As for the anatomical sites irradiated by PBM, the cheek mucosa, upper and lower lips, lateral tongue, and bottom of the mouth stood out. This analysis highlights an increasing interest in PBM as a supportive treatment in cases of OM, as well as the evolution of the technique, types of laser devices, and protocols used.

## 1. Introduction

Oral mucositis [OM] is a common adverse clinical condition in patients undergoing antineoplastic therapies, such as chemotherapy, head and neck radiotherapy, and hematopoietic cell transplantation [1]. The clinical presentation of this condition consists of burning, erythema, and edema of the mucosa, which can progress to ulceration along with painful symptoms, most commonly in the non-keratinized mucosa of the bottom part of the mouth, tongue and soft palate, which can interfere with the routine actions of patients and may even cause the interruption of the anti-cancer treatment [2].

Radiotherapy and chemotherapy lead to the generation of reactive oxygen species, which in turn activate several signaling pathways in the submucosa and epithelium, in addition to triggering damage to the DNA and non-DNA strands of these cells [3]. This pathophysiology activates transcription factors such as nuclear factor kB and an increasing production of pro-inflammatory cytokines such as IL-1b, TNF-a, IL-6, and IL-8 [4], which result in the loss of epithelial cell turnover, apoptosis, atrophy and, as a consequence, the formation of OM. The amplification of these events also occurs through subsequent infection of the compromised mucosa by oral bacteria [5,6].

Several treatments are proposed in the literature to minimize the symptoms of the most severe stages of OM, such as photobiomodulation (PBM). PBM is offered as a non-invasive method, with low risk for the patient and satisfactory results both in the prevention and treatment of OM [7,8,9]. This treatment aims to provide beneficial effects such as analgesia, modulation of the inflammatory process and reduction of edema by promoting cell biostimulation, through the absorption of light energy by endogenous photoreceptors, which results in the activation of energy production by mitochondrial cytochromes, arising from the transmission of electrons, in addition to promoting rapid regeneration of myofibroblasts that originate fibroblasts and growth factors capable of maintaining tissue repair and cytotoxic protection by promoting a reduction in the neutrophil infiltrate and in the expression of cyclooxygenase-2, without compromising the structure, local overheating and mechanical damage to the affected region of the tissue [10,11,12,13].

In recent years, the use of PBM as a preventive and therapeutic method for cases of OM has been frequently investigated. Still, despite this, to the best of our knowledge, bibliometric studies in the area have not been carried out. Bibliometrics is a study that allows the evaluation of scientific publications to identify influential articles in a particular field [9]. It can also be defined as a method that combines science and statistical mathematics, which has numerous advantages, such as the elaboration of a knowledge mapping that results in the understanding of the main methodological changes that occurred in a historical and global context; analyze the impact and scientific growth [11]; identify and discuss possible differences between the proposed protocols and their main findings; indicate research trends and even assist in the creation of public health policies [9,11,12,13].

In this perspective, the aim of the present study was, for the first time, to identify and analyze the literature that relates the association of photobiomodulation with OM, through a bibliometric approach.

## 2. Materials and Methods

Bibliometric studies are a kind of analysis that provides the group of researchers with a broad understanding since it investigates the main ideas, authors, journals, and countries with the highest number of publications in the studied field [14,15].

Considering that a high number of citations indicate the influence of an article on the development of the proposed subject, the parameter commonly used in the bibliometric analysis is citations [14,16,17]. The citation method assesses the frequency that other authors have cited a publication, resulting in its clinical and scientific importance within a specific field of knowledge [18,19,20]. Different indexes, such as the impact factor, are seen as an indirect indicator of quality, productivity and prestige [21,22]. The topics, as well as the methodology of the most cited articles, can encourage future research and, therefore, influence clinical behavior [23]. However, high citation rates can also indicate methodological criticisms or negative results that compromise the study’s credibility. One more factor is the possibility of temporal bias in citations since articles tend to accumulate citations over the years, establishing a knowledge network about the studied topic [24,25,26].

A bibliometric search was carried out on April 27, 2022. The research was conducted at the Thompson Reuters Web of Science citation index database, considering the Web of Science Core Collection (WoS-CC). There were no restrictions to languages or publication years. Editorials, comments, letters, and conference papers were excluded. Scopus and Google Scholar databases were used for subsequent comparisons of the citation number of the selected articles. Two researchers performed the selection of papers and data extraction independently, based on the eligibility criteria. Divergences of opinion were resolved by consensus with a third researcher.

The search strategy followed the protocol recommendation and standardized with the specific terms: TS = (“Mucositides, Oral” OR “Mucositis, Oral” OR “Oral Mucositides” OR “Oral Mucositis” OR Oromucositides OR Oromucositis OR “Oropharyngeal mucositis” OR “ulcerative mucositis” OR “acute oral mucositis” OR “radiation-induced oral mucositis” OR “mucositis secondary to câncer therapy” OR “mucositis induced by oncotheraphy”) AND TS = (“Biostimulation, Laser” OR “Irradiation, Low-Power Laser” OR LLLT OR “Laser Biostimulation” OR “Laser Irradiation, Low-Power” OR “Laser Phototherapy” OR “Laser Therapies, Low-Level” OR “Laser Therapies, Low-Power” OR “Laser Therapy, Low Level” OR “Laser Therapy, Low Power” OR “Laser Therapy, Low-Level” OR “Laser Therapy, Low-Power” OR “Light Therapies, Low-Level” OR “Light Therapy, Low-Level” OR “Low Level Laser Therapy” OR “Low Level Light Therapy” OR “Low Power Laser Irradiation” OR “Low Power Laser Therapy” OR “Low-Level Laser Therapies” OR “Low-Level Laser Therapy” OR “Low-Level Light Therapies” OR “Low-Power Laser Irradiation” OR “Low-Power Laser Therapies” OR “Low-Power Laser Therapy” OR “Photobiomodulation Therapies” OR “Photobiomodulation Therapy” OR “Phototherapy, Laser” OR “Therapies, Low-Level Light” OR “Therapies, Photobiomodulation” OR “Therapy, Low-Level Light” OR “Therapy, Photobiomodulation”).

The ranking of the top 50 most-cited articles was arrayed in descending order, based on their citation count in the database. In the case of drawn, it was considered the high citation density from WoS-CC (i.e., average citation received by an article per year). Articles that analyzed and/or discussed the actions of photobiomodulation in OM were selected as an inclusion criterion. As for the exclusion criteria, some articles did not present OM as the main consequence of the antineoplastic treatment, did not present photobiomodulation as the primary treatment for OM and did not correlate OM and photobiomodulation. All search results were made available in a Microsoft Excel data extraction table. In each publication, the following items were identified: article’s title; year of publication; citation density; authorship; institution and country of origin (based on the address provided for the corresponding authors); journal’s title; impact factor; keywords; study design.

The research results were also imported into the VOS viewer (CWTS, Leiden University, Leiden, Netherlands), a software used to build the collaboration network among authors, including authors with a minimum of 2 documents. The organization of terms was set in groups, with each group representing a color. The most important terms had larger circles and the closely related terms were placed close to each other. Moreover, the lines delineated similarities between the authors, with thicker lines indicating a stronger connection [27,28,29]. Descriptive statistical analyses was performed using the Microsoft Excel program. For the graphical representation of data from countries and continents, MapChart was used (mapchart.net/index.html).

## 3. Results

**Figure 1:**
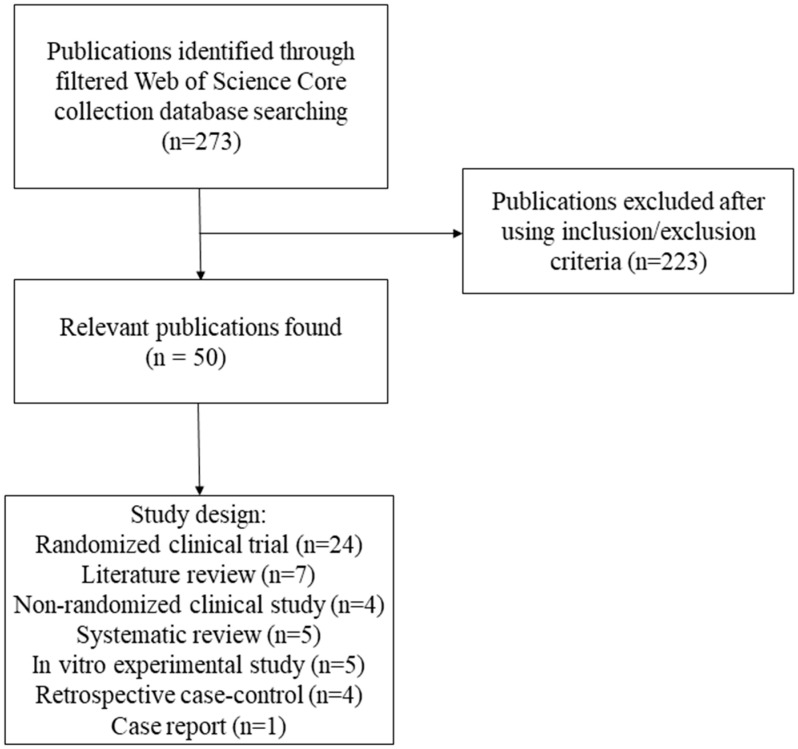
Flowchart of the filtering process.

According to the WoS-CC, 273 publications were found by the search strategy mentioned above. However, 50 articles met the inclusion and exclusion criteria and were analyzed (Figure 1). The ranking of the top 50 most-cited articles published about oral mucositis and photobiomodulation was based on the highest number of citations in WoS-CC (Supplementary Table S1). The top 50 most-cited articles received a total of 6158 (WoS-CC); 2920 (Scopus) and 6255 (Google Scholar) citations. The first most-cited article in the three cited databases (WoS-CC = 157; Scopus = 204; Google Scholar = 367) was “Low-energy He Ne laser in the prevention of radiation-induced mucositis—A multicenter phase III randomized study in patients with head and neck cancer” [30]. The second most cited article (WoS-CC = 150; Scopus = 188; Google Scholar = 322) was “Low energy helium-neon laser in the prevention of oral mucositis in patients undergoing bone marrow transplant: Results of a double-blind randomized trial” [31]. The third most-cited article received 149 citations on WoS-CC and 338 citations on Google Scholar. It was entitled as “A systematic review with meta-analysis of the effect of low-level laser therapy (LLLT) in cancer therapy-induced oral mucositis” [32]. This last article was not found in the Scopus database.

The oldest paper was from 1997, published in International Journal of Radiation Oncology, Biology, Physics, and it was entitled “Low energy helium-neon laser in the prevention of oral mucositis in patients undergoing bone marrow transplant: Results of a double-blind randomized trial” [31]. The latest paper was from 2019 and was entitled “Systematic review of photobiomodulation for the management of oral mucositis in cancer patients and clinical practice guidelines” [33]. It was published on Supportive Care in Cancer.

Randomized clinical trial (48%; 1576 citations on WoS-CC; 1628 on Scopus; 3280 on Google Scholar), followed by Literature review (14%; 462 citations on WoS-CC; 457 on Scopus; 903 on Google Scholar) were the most common study design. Non-randomized clinical study (213 citations on WoS-CC; 208 on Scopus; 452 on Google Scholar), systematic review with meta-analysis (417 citations on WoS-CC; 263 on Scopus; 884 on Google Scholar), in vitro experimental study (214 citations on WoS-CC; 171 on Scopus; 418 on Google Scholar), retrospective case-control (135 citations on WoS-CC; 94 on Scopus; 252 on Google Scholar), systematic review (93 citations on WoS-CC; 107 on Scopus; 159 on Google Scholar), and case report (28 citations on WoS-CC; 31 on Scopus; 69 on Google Scholar) were 10%, 8%, 8%, 8%, 2% and 2%, respectively, the common study design of the 50 most cited papers.

A total of 25 journals published the top 50 most cited publications. The one with the most publications was Supportive Care in Cancer (20%; 1000 citations on WoS-CC; 951 citations on Scopus; 2002 citations on Google Scholar), followed by Oral Oncology (10%; 285 citations on WoS-CC; 178 citations on Scopus; 530 citations on Google Scholar), Photomedicine and Laser Surgery (8%; 196 citations on WoS-CC; 211 citations on Scopus; 396 citations on Google Scholar), Lasers in Medical Science (8%; 158 citations on WoS-CC; 114 on Scopus; 311 citations on Google Scholar) and Lasers in Surgery and Medicine (6%; 172 citations on WoS-CC; 183 citations on Scopus; 302 citations on Google scholar). The impact factors for journals with the top 50 cited articles ranged from 1.28 (Journal of Pediatric Hematology Oncology) to 22.11 (Blood). These two journals presented most publications related to advances in diagnosing and treating cancer and blood diseases in children and hematology in general, respectively.

**Figure 2:**
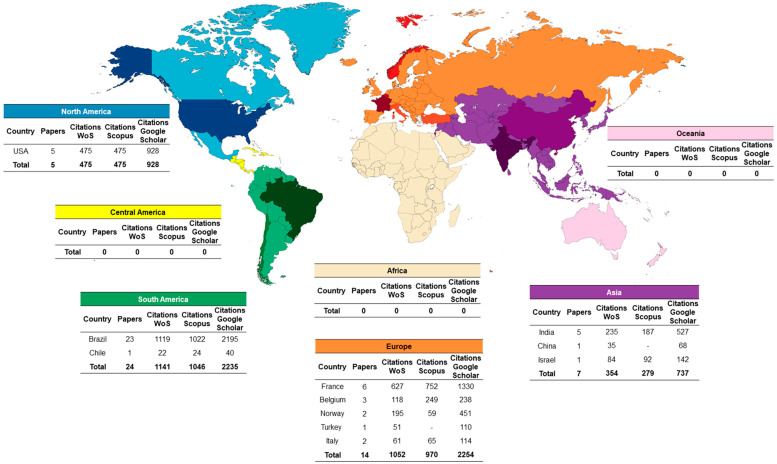
Worldwide distribution**.** The 50 topmost cited articles about photobiomodulation and oral mucositis.

There were 11 different countries of origin: Brazil had the highest number of articles (46%; 1141 citations on WoS-CC; 1153 on Scopus; 2231 on Google Scholar), followed by France (12%; 634 citations on WoS-CC; 768 on Scopus; 1335 on Google Scholar), United States (10%; 482 citations on WoS-CC; 480 on Scopus; 944 on Google Scholar), India (10%; 238 citations on WoS-CC; 221 on Scopus; 535 on Google Scholar), Belgium (6%; 120 citations on WoS-CC; 94 on Scopus; 249 on Google Scholar), Norway (4%; 198 citations on WoS-CC; 59 on Scopus; 454 on Google Scholar), Italy (4%; 61 citations on WoS-CC; 66 on Scopus; 123 on Google Scholar), Israel (2%; 93 citations on WoS-CC; 107 on Scopus; 159 on Google Scholar), Turkey (2%; 52 citations on WoS-CC; 110 on Google Scholar), China (2%; 37 citations on WoS-CC; 76 on Google Scholar) and Chile (2%; 23 citations on WoS-CC; 24 on Scopus; 43 on Google Scholar). South America (50%) was the continent with most papers on the 50 most cited list, followed in sequence by Europe (28%), Asia (14%) and North America (10%). The first author’s country was taken as the country of the article’s origin. Central America, Africa and Oceania did not have countries on the cited list (Figure 2).

**Figure 3:**
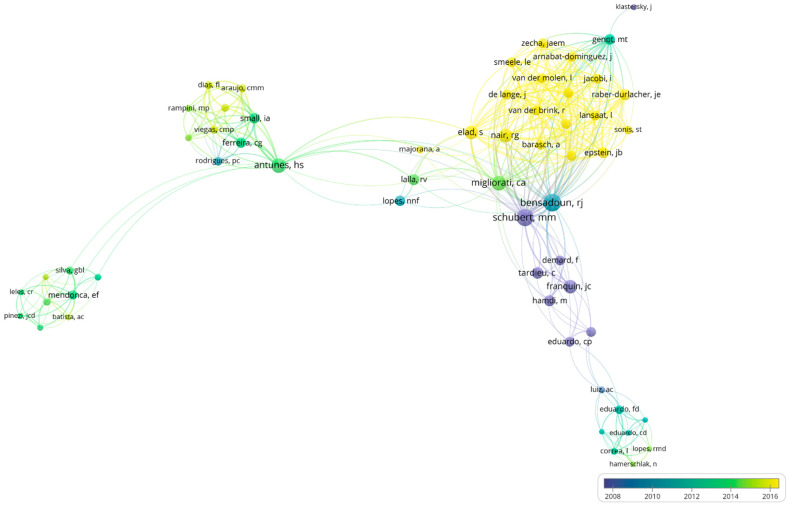
Network visualization per author**.** Bibliographic coupling between the most-cited authors in the top 50 most-cited papers.

The results of the author analysis have identified 260 researchers. The most-cited authors were Bensadoun with 8 (16%) published articles (3.1%; 837 citations on WoS-CC), Schubert with 6 (12%) published articles (2.9%; 774 citations on WoS-CC), Migliorati with 5 (10%;) published articles (2%; 551 citations on WoS-CC), Antunes with 7 (14%) published articles (1.9%; 520 citations on WoS-CC), Franquin with 3 (6%) published articles (1.6%; 437 citations on WoS-CC), Nair 5 (10%) published articles (1.6%; 423 citations on WoS-CC) and Elad with 4 (8%) published articles (1.6%; 421 citations on WoS-CC). VOSviewer map was built to verify bibliometric coupling and the connection between authors (Figure 3).

The image below depicted and detailed the existence of connections between the co-authorship. The primary connections and high density of citations contained prominent authors, such as Bensadoun, Schubert and Franquin, bringing together other collaborators who had few networks of relationships between them. Still, a collaborative network was perceived among all authors. It was noticed that the older the author’s publication, the greater their citation network number was.

Among the institutions network, a total of 125 institutions contributed, with 14,986 citations on WoS-CC, when considering authors and co-authors. The most significant contribution was made by the University of Sao Paulo, Brazil (4.4%; 670 citations on WoS-CC), followed by Fred Hutchinson Centre Research Institute, USA (2.9%; 437 citations on WoS-CC), University of Rochester, USA (2.8%; 421 citations on WoS-CC), National Cancer Institute, Brazil (2.2%; 339 citations on WoS-CC) and Ctr Antoine Lacassagne, France (2.2%; 337 citations on WoS-CC).

**Figure 4:**
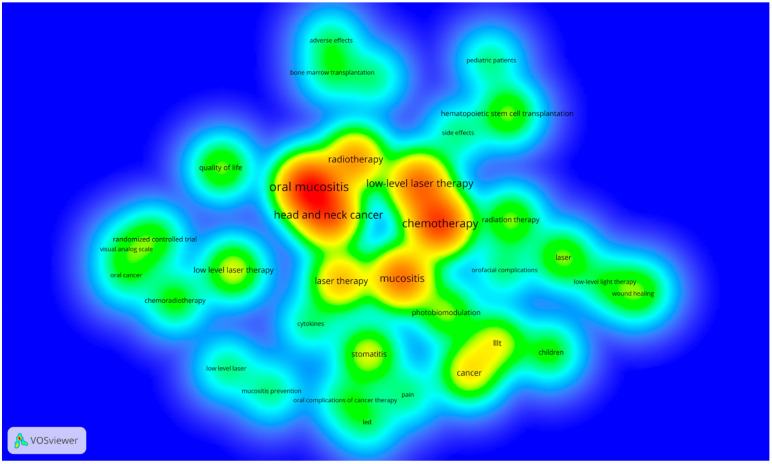
Keywords density map**.** The most frequent keywords of the 50 most-cited articles. Colors indicate the citation density of authors, ranging from blue (lowest density) to red (highest density).

Of the 50 top-cited articles, 235 keywords were identified (Figure 4). The most frequent were chemotherapy (4.7%; n = 30), followed by oral mucositis (3.4%; n = 22), prevention (3.1%; n = 20), helium-neon laser (2.8%; n = 18), radiotherapy (2.8%; n = 18), radiation-induced mucositis (2.3%; n = 15), head and neck cancer (2.1%; n = 14) and mucositis (2.1%; n = 14). These most cited keywords can help elucidate the most searched points of interest in the evaluated topic.

Table 1 demonstrates the main findings of the application protocols of photobiomodulation on OM considering clinical, observational and experimental papers (76%; n = 38). The anatomical site that is irradiated by PBM are cheek mucosa (78.9%; n= 30), upper and lower lip (76.3%; n = 29), lateral of the tongue (68.4%; n = 26), the bottom of mouth (60.5%; n = 23), soft palate (47.3%; n = 18), ventral of the tongue (44.7%; n = 17), oropharynx (15.7%; n = 6), dorsal of the tongue (13.1%; n = 5), lip commissure (10.5%; n = 4), hard palate (7.8%; n = 3), gum (5.2%; n = 2), uvula (5.2%; n = 2), palatine tonsil (5.2%; n = 2) and retromolar region(2.6%; n = 1). About the protocol objective, treatment (52.6%; n = 20) was the principal followed by prevention (44.7%; n = 17). The PBM application frequency was indicated as twice a week (2.6%; n = 1), three times a week (7.8%; n = 3), four times a week (2.6%; n = 1), five times a week (57.8%; n = 22) and not specified (21%; n = 8). When considering the main anti-cancer therapy, chemotherapy (63.1%; n = 24), was the first, followed by radiotherapy (44.3%; n = 18) and bone marrow transplant (26.3%; n = 10).

**Figure 5:**
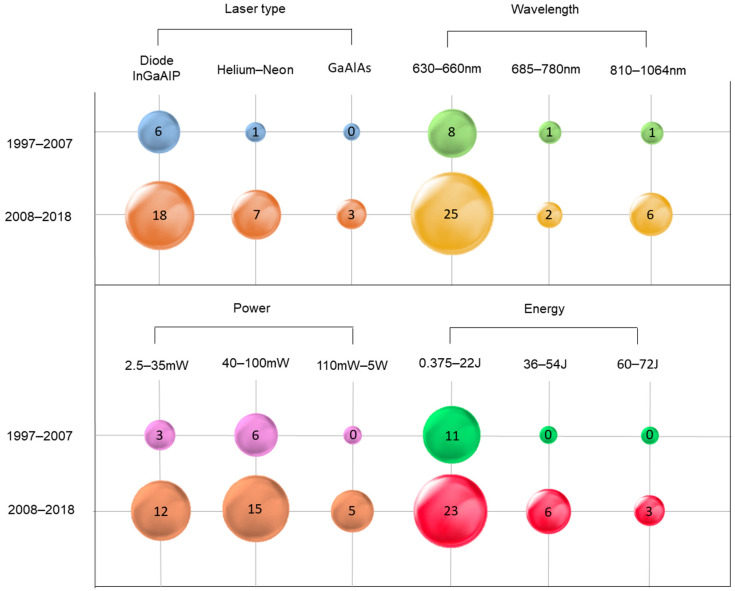
Main specifications of the lasers used in the studies. The number inside each circle indicates the number of articles with the evaluated parameters: type of laser, wavelength, power and energy.

The main specifications of the lasers used in the studies, considering clinical, observational and experimental papers (76%; n = 38), are shown in Figure 5. In the decade of 1997 to 2007, the main laser type used was InGaAlP diode (Indium-Gallium-Aluminum Phosphide) (15.7%; n = 6), followed by Helium-Neon (He-Ne) (2.6%; n = 1), the wavelength was ranged in 630–660 nm (21%; n = 8), 685–780 nm (2.6%; n = 1) and 810–1064 nm (2.6%; n = 1), the power was ranged in 2.5–35 mW (7.89%; n = 3), 40–100 mW (15.7%; n = 6), and the energy was ranged in 0.375–22 J (28.9%; n = 11). In the decade of 2008 to 2018, the main laser type used was InGaAlP diode (47.3%; n = 18), followed by He-Ne (18.4%; n = 7) and Gallium and Aluminum Arsenide (GaAlAS) (7.89%; n = 3), wavelength was ranged in 630–660 nm (65.7%; n = 25), 685–780 nm (5.2%; n = 2) and 810–1064 nm (15.7%; n = 6), the power was ranged in 2.5–35 mW (31.5%; n = 12), 40–100 mW (39.4%; n = 15) and 110 mW–5 W (13.1%; n = 5). Additionally, the energy ranged in 0.375–22 J (60.5%; n = 23), 36–54 J (15.7%; n = 6) and 60–72 J (7.89%; n = 3).

## 4. Discussion

In the present study, quali-quantitative analyses were performed on the 50 most cited articles associated with the terms “photobiomodulation” and “oral mucositis.” It was observed that clinical, observational, and experimental papers that evaluated the action of PBM on OM used different types of protocols. The most cited anatomical sites irradiated by PBM were cheek mucosa, upper and lower lips, lateral tongue, back of the mouth, soft palate, ventral tongue, oropharynx, dorsal tongue, and labial commissure. Treatment was the primary goal of the protocol, followed by prevention. The frequency of PBM application was used twice a week, three times a week, four times a week, and five times a week and not specified. Regarding the primary anti-cancer therapy, chemotherapy was the first, followed by radiotherapy and bone marrow transplant. The main laser type used was InGaAlP diode, the wavelength ranged from 630–660 nm, the power ranged from 40–100 mW, and the energy density ranged from 0.375–22 J/cm^2^.

The central database used in this study was WoS-CC, while Scopus and Google Scholar were used for comparison purposes. However, the database with the highest number of citations was Google Scholar, previously demonstrated by other bibliometric studies [11,23,32]. Considering that this database includes citations from books, theses, dissertations, and open-access online journals [24]. Therefore, this result is within what was expected [34,35,36].

The impact factor is an index to classify the prestige and reliability of scientific journals. It is calculated based on the annual average of citations according to the performance of scientific articles, their authors, journals, or institutions. It demonstrates the relevance that the journal and its scope have in contributing to the field of general or specific scientific research. Because it is a mathematical classification metric, the higher the journal’s index, the better. As a result, the journal is more selective in accepting manuscripts for publication [37,38].

The journals included in this bibliometric study have an average impact factor of 4.25, among which only one had a much higher impact value than the others (Blood: 22.11), a publication of The American Society of Hematology. The article was published in this journal under a specific scientific category called Transplantation, demonstrating the relevance of studying the subject, including in transplant patients. Among the 50 most cited articles, the journal Supportive Care in Cancer (impact factor = 3.603) had the highest number of published articles. In our bibliometric survey, the studies published in this journal comprise 4 randomized clinical trials, 3 systematic reviews, 2 literature reviews, and 1 retrospective case–control.

The frequency of publication of articles on photobiomodulation and OM has increased in recent years, using Cowen’s 1997 study as an initial comparison [39]. Our data show that, between 1997 and 2007, 10 articles were published, with a total of 782 citations in WoS-CC. It is noteworthy that, at that time, the most used type of laser was InGaAlP diode type, followed by He-Ne, with a wavelength of 632.8 nm and power ranged from 2.5–35 mW and energy density ranged from 0.375–22 J/cm^2^ [34,39].

Despite the increase in the variety of laser types in the second decade (2008–2019), the diode remains the most used among the others, probably because it is a low-cost device compared to He-Ne and GaAlAS type, portable, and easy to handle. Moreover, this type of laser delivers the power 40 mW or 100 mW and has two wavelengths, 660 nm for red and 808 nm for infrared. Concerning power, Simões et al. 2009 [5] analyzed the difference between low and high power in head and neck cancer patients undergoing radiotherapy. An InGaAlP diode laser was used, with a wavelength of 660 nm and power of 40 mW, being used alone and in conjunction with a GaAlAs laser with a wavelength of 808 nm and 1 W of power, noting that the combination of powers brought more significant benefits to the patients, maintaining the severity of OM in I to II on OMS level. However, satisfactory results were obtained when the low power was used alone compared to the high power. Antunes et al. [40,41] analyzed the preventive effect of PBM in patients with oral mucositis who had received radiotherapy. The preventive application of the laser was performed daily, five times a week, during the entire antineoplastic treatment period. The laser was applied punctually in contact with the mucosa in nine points, 0.24 cm^2^, per region for 10 s in each point, totaling 12 min of exposure. The total energy density used was 4 J/cm^2^.

Interestingly, the results of our study demonstrate that most of the 50 top-cited articles originated from Brazil, unlike other bibliometric studies previously published in other areas of dentistry [11,14]. The main institutions are the University of São Paulo and the Instituto Nacional do Câncer, which presented comparative clinical studies regarding the applicability protocol of PBM when compared to placebo, as well as its preventive effect and its photobiomodulatory action.

It is valid to note that the oldest articles published by the University of São Paulo were from 2009 [5], which compared the effect of low-level versus high-level photobiomodulation, and the association between them, in patients undergoing radiotherapy in the head and neck. The low-power laser used was the InGaAlP diode type, with the wavelength of 660 nm, power of 40 mW, the energy density of 6 J/cm^2^ and energy per point of 0.24 J, with 24 total points that received laser radiation intraorally. As for high power, the GaAlAs laser was used, with a wavelength of 808 nm, power of 1 W/cm^2^ and energy density of approximately 10 J/cm^2^ in the same 24 points in the oral cavity were irradiated at low power. The results of this study demonstrate that the low-power laser alone or associated with the high-power laser applied three times a week showed that the OM degrees remained at the maximum levels of I and II. In addition, its applicability also avoided the increase in pain that resulted in the absence of unplanned interruptions of anti-cancer treatment, reducing the length of stay and significant costs for the hospital.

The most recent was from 2015, a case–control study with pediatric patients who received previous oral hygiene care and PBM during bone marrow transplant treatment. The laser used was the InGaAIP diode type, 660 nm, power of 100 mW, the energy density of 8 J/cm^2^, 8 s per point, and energy per point of 0.24 J, and 16 points of the oral cavity were selected to be irradiated [42]. It was concluded that young patients analyzed in this study who underwent daily specialized oral care and PBM had mild degrees of OM during transplantation. In conclusion, dental care before bone marrow transplantation and PBM is effective and beneficial for the patient’s well-being and prevents the emergence of severe OM.

Different principal authors published the two studies mentioned above, but with the same network of co-authors and study groups, thus noting the similarity in the parameters of the low-power device that was used, as well as in the application points, in addition to demonstrating the heterogeneity methodological approach that the research group works in the context of photobiomodulation. Regarding the National Cancer Institute, the oldest article was from 2007 [3], and the most recent was from 2017 [40], both by the same author.

The main focus of the studies included in the 50 most cited articles was the preventive and therapeutic effect of photobiomodulation on oral mucositis in cancer patients undergoing chemotherapy [6,40,41,43,44,45,46,47,48,49,50,51], hematopoietic cell transplantation [3,31,48,52,53,54,55] and head and neck radiotherapy [5,30,49,56,57,58]. Most articles are clinical intervention studies, being randomized and non-randomized, in which photobiomodulation and its analgesic action are compared with patients who did not receive any intervention. [40,42,49,52]. It can be seen that, over the years, the type of laser varied from He-Ne [4,30,31,47,58,59], GaAlAS [60,61] to the most used, diode InGaAlP [3,4,5,6,40,41,43,44,45,48,49,50,51,52,53,54,56,62,63,64,65].

It was observed that anatomical points such as oral mucosa, upper and lower lips, lateral of the tongue, and bottom of the mouth are commonly cited regions in articles for PBM radiation both as a preventive and as a treatment, provided they are in the opposite direction to the tumor when located in the head and neck region [5,6,43,45,48,49,51,53,54,55,56,62,63,64]. In addition, the literature demonstrates that non-keratinized epithelial mucosa is prone to develop severe OM according to the anti-cancer treatment regimen regarding the restorative action in cases of oral mucositis, which was evaluated by an established score [66].

Among the most cited studies, it is worth mentioning one that analyzed the effect of photobiomodulation as a preventive measure for OM in the posterior third of the internal surfaces of the cheeks, soft palate, and anterior tonsillar pillars in patients undergoing head and neck radiotherapy. The He-Ne laser was irradiated intraorally, with a wavelength of 632.8 nm, 60 mW, and 25 mW of power, according to where the patients were treated (Nice, Marseilles, and Reims, respectively). The authors mentioned the difficulties of using the laser in this region [30].

Subsequently, another study [47] analyzed the action of photobiomodulation on OM measurements and subjective quality of life outcomes after chemoradiotherapy in patients with head and neck cancer. For this purpose, a He-Ne laser irradiation protocol was used, wavelength 632.8 nm, power density 24 mW/cm^2^, dosage 3.0 J at each point, and total dose/session 36–40 J. Irradiation was performed on the borders of the tongue, floor of the mouth, buccal mucosa, labial mucosa, soft palate and oropharynx. The results of this study demonstrated that photobiomodulation effectively improved the subjective experience of OM and the quality of life of these patients.

Only one study compared different types of lasers (InGaAlP and GaAlAs), wavelength (660, 810, 980, and 1064 nm), and power (100 mW and 0.25 W) on the healing of mucositis by evaluating the expression of platelet-derived growth factor (PDGF), transforming growth factor beta (TGF-β) and blood-derived fibroblast growth factor (bFGF) in an animal model. The type of laser used in the wavelengths 660 nm, 810, and 980 nm, and 8 J/cm^2^, 8.3 J/cm^2^ and 8 J/cm^2^ of energy density, respectively, and power of 100 mW, was an InGaAlP diode. Additionally, the type of laser used in the wavelengths 1064 nm, 8 J/cm^2^ of energy density, and 0.25 W of power was a GaAlAs. The study suggests that 980 nm diode laser therapy and low-level Nd:YAG obtained better results when compared to the others since they promoted the acceleration of the wound healing process by modifying the expression of the PDGF and bFGF genes, which are responsible for stimulating cell proliferation and fibroblast growth. [61].

Several articles used PBM with different numbers of weekly applications, for example, on alternate days, either twice a week [49,52] or 3 times a week [43]. However, most of the articles analyzed used PBM 5 times a week on consecutive days, justifying that the daily stimulus promotes a rapid response in the production of pro-inflammatory cytokines, resulting in the improvement of the inflamed region. Therefore, the daily application of PBM has become standard in the literature [3,5,6,30,31,40,41,42,44,48,50,54,55,56,62,64,65]. Unfortunately, no studies compared the different types of application protocols nor the frequency of application.

The Multinational Association of Supportive Care in Cancer and International Society for Oral Oncology (MASCC/ISOO), National Institute for Health and Care Excellence (NICE), and Walt Working Group developed guides to propose established protocols for the use of PBM in the OM preventively and treatment [67,68,69]. These guides coincide in terms of device calibrations such as wavelength, power density, exposure time of each anatomical point, and the frequency of the session used. Among the studies on selected human beings, only three used the protocol recommended by such guides [43,49,52]. However, it should be noted that among the selected articles, several were published before the first protocol launched in 2004 by MASCC/ISOO [30,31,44]. As for those published after the last update of this study group, as well as NICE, variation was observed in the frequency of use of photobiomodulation, anatomical points, and power density, thus demonstrating the absence of the use of these protocols established in the literature. Despite that, all clinical studies have shown promising results regarding the use of low-frequency PBM and potency for the prevention of severe OM and the treatment of this clinical condition. It is worth mentioning that MASCC/ISOO, NICE, and the Walt Working Group do not establish specific anatomical points to be irradiated by PBM, also observing the wide variability of points that the studies used.

The articles published between 1997 and 2007 are mostly clinical trials that aimed to analyze the photobiomodulatory effect of PBM on oral mucositis in patients undergoing anti-cancer treatment. [3,30,31,43,44,45,52,53,57]. Regarding those published between 2008 and 2019, there was an increase in the variety of types of studies, with the presence of literature reviews aiming to contextualize the history, potential action, types of devices, and photobiomodulation functionalities [64,70], reviews, systematic reviews that discuss the management and treatment protocol of therapy in patients with mucositis [32,39], and a systematic review that presents a proposal for a guideline for the management of those patients [33]. The number of experimental studies in vivo with models of oral mucositis in rodents has also increased, and photobiomodulation as the main treatment proposal, varying the wavelengths of the low-power device and its anti-inflammatory effect. [4]. However, most studies continue to be in humans, such as observational studies that aim to analyze the influence of adding photobiomodulation in the hospital oncology service [42] and clinical trials, which analyzed different types of protocols and laser devices [4,40,41,43,44,45,46,47,48,54,62,63].

In this context, when checking the overview of the 100 most cited articles in Dentistry [71] in the period from 1955 to 2014, it was observed that the most frequent study designs were literature review (36%) and clinical trial (24%), so these findings are partially correlated with the results of the present study, considering that the topic of photobiomodulation and mucositis is contemporary, with emphasis on the publication of articles from the 1990s onwards.

All 260 authors who contributed to the 50 most cited articles were involved in at least two, either as primary authors or co-authors. The author Bensadoun was the one who contributed the most and thus presented the highest citation among the selected authors. The articles with the lowest number of authors (n = 2) were two literature reviews [59,72] and one clinical study from Europe [43]. Interestingly, the article with the highest number of authors (n = 21) came from Europe and was a literature review [67].

Regarding the demographic distribution of the authors, the results of our analysis showed that the first authors and the co-authors from South America made significant contributions to the 50 most cited articles. This result was different from what was shown in previous studies published in other areas of dentistry and medicine, which showed a small contribution from authors in these fields. [11,58,73,74]. It is noteworthy that the author with the greatest contribution from South America was Antunes, with strong citation power. Self-citations were included in this study, as authors who routinely work in specific research are likely to cite their articles for comparison purposes. Self-citations are considered practices of great contribution by authors in their field of knowledge [23].

## 5. Conclusions

To the best of our knowledge, this is the first study to carry out an objective and quantitative bibliometric analysis of the 50 most cited articles about the effect of photobiomodulation on OM. Among these, most were about clinical intervention, and their main focus was the use of photobiomodulation in the prevention and treatment of OM in cancer patients. South America and Brazil were the continent and country with the highest number of articles, respectively. Furthermore, it was observed that, after 2011, there was a significant increase in the number of published articles, which demonstrates the interest in the subject, especially regarding establishing the most appropriate treatment protocol to ensure better patient comfort and well-being.

## Figures and Tables

**Table 1:** The main findings of the application protocols of photobiomodulation on OM.

Protocol Characteristics	Absolute Frequency	Relative Frequency (%)
**Anatomical site**		
Cheek mucosa	30	78.9
Upper and lower lip	29	76.3
Lateral of the tongue	26	68.4
Bottom of mouth	23	60.5
Soft palate	18	47.3
Ventral of the tongue	17	44.7
Oropharynx	6	15.7
Dorsal of the tongue	5	13.1
Lip commissure	4	10.5
Hard palate	3	7.8
Gum	2	5.2
Uvula	2	5.2
Palatine tonsil	2	5.2
Retromolar region	1	2.6
**Protocol objective**		
Treatment	20	52.6
Prevention	17	44.7
**PBM session application**		
Twice a week	1	2.6
Three times a week	3	7.8
Four times a week	1	2.6
Five times a week	22	57.8
Not specified	8	21
**Anti-cancer treatment**		
Chemotherapy	24	63.1
Radiotherapy	18	44.3
Bone marrow transplant	10	26.3

PBM: photobiomodulation.

## Data Availability

All data is available in the main text and Supplementary Material.

## Supplementary Materials

The following supporting information can be downloaded at: https://www.mdpi.com/article/10.3390/life12111940/s1. Table S1: Effects of photobiomodulation on oral mucositis: visualization and analysis of knowledge.
