# Supplementary material for: Effects of Photobiomodulation on Oral Mucositis: Visualization and Analysis of Knowledge
Source: Life (Basel). 2022 Nov 21;12(11):1940. doi: 10.3390/life12111940 (PMC9694968; doi:10.3390/life12111940)
Supplement: Supplementary file 1 [file life-12-01940-s001.zip › life-1932369-supplementary.pdf]

Supplementary Table S1. The 50 most cited papers on the effect of photobiomodulation on oral mucositis

Rank	Article Title	Author Name	Times Cited (Citation average)			DOI	Study design	Abstract
			WoS-CC	Scopus	Google Scholar			
1	Low-energy He Ne laser in the prevention of radiation-induced mucositis - A multicenter phase III randomized study in patients with head and neck cancer	Bensadoun, RJ. et al. (1999)	157 (7,1)	204 (9,1)	373 (16,9)	10.1007/s005209900034	Randomized clinical trial	Evaluation of the use of low-level laser as prevention of oral mucositis in patients with head and neck cancer. Multicenter, randomized phase III study.
2	Low energy helium-neon laser in the prevention of oral mucositis in patients undergoing bone marrow transplant: Results of a double blind randomized trial	Cowen, D. et al. (1997)	150 (6,2)	188 (7,8)	322 (13,4)	10.1016/s0360-3016(97)00076-x	Randomized clinical trial	Evaluation of low energy helium-neon laser in the prevention of oral mucositis in patients undergoing bone marrow transplantation.
3	A systematic review with meta-analysis of the effect of low-level laser therapy (LLLT) in cancer therapy-induced oral mucositis	Bjordal, JM. et al. (2011)	149 (14,6)	-	338 (30,5)	10.1007/s00520-011-1202-0	Systematic Review with Meta-analysis	Systematic review addressing the action of low-level laser therapy in oral mucositis therapy.
4	Systematic review of laser and other light therapy for the management of oral	Migliorati, C. et al. (2013)	136 (16,7)	162 (19,8)	275 (32,6)	10.1007/s00520-012-1605-6	Systematic Review with Meta-	Systematic review addressing the use of low-level laser therapy as a therapy for oral mucositis.

	mucositis in cancer patients						analysis	
5	A phase III randomized double-blind placebo-controlled clinical trial to determine the efficacy of low-level laser therapy for the prevention of oral mucositis in patients undergoing hematopoietic cell transplantation	Schubert, MM. et al. (2007)	132 (9,2)	160 (11,3)	275 (19,3)	10.1007/s00520-007-0238-7	Randomized clinical trial	Randomized clinical trial evaluating the efficacy of low level laser therapy in the treatment of oral mucositis in hematopoietic cell transplant patients.
6	Low-level laser therapy/photobiomodulation in the management of side effects of chemoradiation therapy in head and neck cancer: part 2: proposed applications and treatment protocols	Zecha, JAEM. et al. (2016)	109 (21,2)	123 (23,4)	180 (34,6)	10.1007/s00520-016-3153-y	Literature review	Literature review with a proposal for a protocol for the application of low level laser therapy in the treatment of oral mucositis in patients undergoing chemotherapy and radiotherapy.
7	Low level laser therapy/photobiomodulation in the management of side effects of chemoradiation therapy in head and neck cancer: part 1: mechanisms of action, dosimetric, and safety considerations	Zecha, JAEM. et al. (2016)	100 (19,4)	111 (21,2)	171 (32,8)	10.1007/s00520-016-3152-z	Literature review	Literature review that addresses the mechanism of action, dosimetry and safety issues of low-level laser therapy in the treatment of oral mucositis in patients with head and neck cancer.
8	Low-power laser in the prevention of induced oral mucositis in bone marrow transplantation patients: a randomized trial	Antunes, HS. et al. (2007)	97 (6,9)	107 (7,6)	180 (12)	10.1182/blood-2006-07-035022	Randomized clinical trial	Randomized clinical study evaluating low-level laser therapy as a treatment for oral mucositis in bone marrow transplant patients.

9	Effect of Prophylactic Low Level Laser Therapy on Oral Mucositis: A Systematic Review and Meta-Analysis	Oberoi, S. et al. (2014)	95 (13,2)	101 (14,2)	195 (26,7)	10.1371/journal.pone.0107418	Systematic Review with Meta-analysis	Systematic review with meta-analysis that addresses the prophylactic use of low-level laser therapy in the prevention of oral mucositis.
10	Systematic review of photobiomodulation for the management of oral mucositis in cancer patients and clinical practice guidelines	Zadik, Y. et al. (2019)	93 (42)	107 (46)	159 (71)	10.1007/s00520-019-04890-2	Systematic Review	Systematic review that addresses low-level laser therapy guidelines in the treatment of oral mucositis.
11	Laser Phototherapy as Topical Prophylaxis Against Head and Neck Cancer Radiotherapy-Induced Oral Mucositis: Comparison Between Low and High/Low Power Lasers	Simoes, A. et al. (2009)	70 (5,8)	77 (6,2)	134 (11,1)	10.1002/lsm.20758	Randomized clinical trial	Randomized clinical study comparing low-level laser therapy with high-frequency laser therapy in the treatment of oral mucositis.
12	Could the biological robustness of low level laser therapy (Photobiomodulation) impact its use in the management of mucositis in head and neck cancer patients	Sonis, ST. et al. (2016)	70 (13,8)	-	102 (19,8)	10.1016/j.oraloncology.2016.01.005	Literature review	Literature review evaluating the photobiomodulatory action of low-level laser therapy in the treatment of oral mucositis.
13	Low-level laser therapy in the prevention and treatment of cancer therapy-induced mucositis: 2012 state of the art based on literature review and meta-analysis	Bensadoun, RJ. et al. (2012)	68 (7,4)	85 (9,2)	155 (15,3)	10.1097/CCO.0b013e328352eaa3	Literature review	Literature review evaluating low-level laser therapy in the prevention and treatment of oral mucositis.

14	Phase III trial of low-level laser therapy to prevent oral mucositis in head and neck cancer patients treated with concurrent chemoradiation	Antunes, HS. et al. (2013).	68 (8,3)	73 (8,8)	113 (12,3)	10.1016/j.radonc.2013.08.010	Randomized clinical trial	Randomized clinical study evaluating low-level laser therapy in the prevention of oral mucositis in head and neck cancer patients undergoing chemotherapy and radiotherapy.
15	Oral mucositis prevention by low-level laser therapy in head-and-neck cancer patients undergoing concurrent chemoradiotherapy: a phase III randomized study	de Lima, AG. et al. (2012)	65 (7,2)	69	118 (12,8)	10.1016/j.ijrobp.2010.10.012	Randomized clinical trial	Randomized clinical study that evaluating low-level laser therapy in the prevention of chemotherapy-induced oral mucositis in head and neck cancer patients.
16	Low-level Infrared Laser Therapy in Chemotherapy-induced Oral Mucositis A Randomized Placebo-controlled Trial in Children	Kuhn, A. et al. (2009)	65 (5,2)	75 (5,9)	148 (11,8)	10.1097/MPH.0b013e318192cb8e.	Randomized clinical trial	Randomized clinical study that evaluating low-level laser therapy in the treatment of chemotherapy-induced oral mucositis in pediatric patients.
17	Effect of low level helium-neon (He-Ne) laser therapy in the prevention & treatment of radiation induced mucositis in head & neck cancer patients	Maiya, GA. et al. (2006)	63 (4,2)	-	155 (10,2)	-	Randomized clinical trial	Randomized clinical study that evaluating low-level laser therapy for the prevention and treatment of oral mucositis in head and neck cancer patients.
18	The Prevention of Induced Oral Mucositis with Low-Level Laser Therapy in Bone Marrow Transplantation Patients: A Randomized Clinical Trial	Silva, GBL. et al. (2011)	63 (6,2)	64 (6,3)	108 (10,8)	10.1089/pho.2009.2699	Randomized clinical trial	Randomized clinical trial evaluating low-level laser therapy in the prevention of oral mucositis in hematopoietic cell transplant patients.
19	Evaluation of low-level laser therapy in the prevention and	Carvalho, PAG. et al.	62 (6,2)	70 (6,9)	141 (14,1)	10.1016/j.oraloncology.20	Randomized	Randomized clinical study that evaluating low-level laser therapy in the prevention

	treatment of radiation-induced mucositis: A double-blind randomized study in head and neck cancer patients	(2011)				11.08.021	clinical trial	and treatment of radiotherapy-induced oral mucositis in head and neck cancer patients.
20	Low-level laser therapy in the prevention and treatment of chemotherapy-induced oral mucositis in young patients	Abramoff, MMF. et al. (2008)	62 (4,7)	70 (5,3)	138 (9,8)	10.1089/pho. 2007.2144	Randomized clinical trial	Randomized clinical trial that evaluating low-level laser therapy in the prevention of oral mucositis.
21	Influence of low-energy laser in the prevention of oral mucositis in children with cancer receiving chemotherapy	Cruz, LB. et al. (2007)	61 (4,2)	70 (4,7)	143 (10)	10.1002/pbc. 20943	Randomized clinical trial	Randomized clinical study evaluating low-level laser therapy in the prevention of oral mucositis in patients undergoing head and neck radiotherapy.
22	Effect of low-level laser therapy on patient reported measures of oral mucositis and quality of life in head and neck cancer patients receiving chemoradiotherapy-a randomized controlled trial	Gautam, AP. et al. (2013)	57 (6,8)	62 (7,5)	120 (14,3)	10.1007/s00 520-012-1684-4	Randomized clinical trial	Randomized clinical study that evaluating low-level laser therapy and the quality of life of patients with head and neck cancer undergoing chemotherapy and radiotherapy.
23	Low level laser therapy for concurrent chemoradiotherapy induced oral mucositis in head and neck cancer patients - A triple blinded randomized controlled trial	Gautam, AP. et al. (2012)	55 (6)	64 (7,1)	125 (13,6)	10.1016/j.rad onc.2012.06. 011	Randomized clinical trial	Randomized clinical study evaluating low-level laser therapy in the treatment of oral mucositis in a patient undergoing chemotherapy and head and neck radiotherapy.
24	Effects of Low-Level Laser Therapy on Collagen	Lopes, NNF. et al. (2010)	54 (4,8)	55 (5)	89 (7,1)	10.1002/lsm. 20920	In vitro experimental	Experimental in vitro study that evaluating the effectiveness of laser therapy on

	Expression and Neutrophil Infiltrate in 5-Fluorouracil-Induced Oral Mucositis in Hamsters						study	collagen expression and treatment of chemically-induced oral mucositis by 5-Fluorouracil in Hamsters.
25	Long-term survival of a randomized phase III trial of head and neck cancer patients receiving concurrent chemoradiation therapy with or without low-level laser therapy (LLLT) to prevent oral mucositis	Antunes, HS. et al. (2017)	53 (12,2)	-	88 (20,5)	10.1016/j.oraloncology.2017.05.018	Randomized clinical trial	Randomized clinical study comparing the action of low-level laser therapy versus the absence of treatment in oral mucositis in patients with head and neck cancer.
26	Effects of laser irradiation at different wavelengths (660, 810, 980, and 1,064 nm) on mucositis in an animal model of wound healing	Usumez, A. et al. (2014)	52 (7,2)	-	110 (15,7)	10.1007/s10103-013-1336-z	In vitro experimental study	Experimental study that evaluating the many different potencies of low-level laser therapy in the treatment of chemically induced oral mucositis in rats.
27	Chemotherapy- and radiotherapy-induced mucositis in head and neck cancer patients: new trends in pathophysiology, prevention and treatment	Bensadoun, RJ. et al. (2001)	50 (2,5)	57 (2,8)	145 (7,2)	10.1007/s004050100368	Literature review	Literature review that evaluating the role of laser therapy as a new treatment for oral mucositis in patients with head and neck cancer undergoing chemotherapy and radiotherapy.
28	Patients with moderate chemotherapy-induced mucositis: pain therapy using low intensity lasers	Nes, AG. (2005)	49 (3)	59 (3,6)	116 (7,1)	10.1111/j.1466-7657.2004.00401.x	Non-randomized clinical study	Clinical study evaluating low-level laser therapy for pain control in oral mucositis in patients undergoing chemotherapy and radiotherapy.
29	Pilot study of laser effects on oral mucositis in patients receiving chemotherapy	Wong, SF. (2002)	49 (2,5)	57 (3)	110 (5,7)	10.1097/00130404-200205000-00008	Pilot clinical study	Clinical study evaluating the effects of low-level laser therapy on oral mucositis in patients undergoing chemotherapy and radiotherapy.

30	Effect of Low-Level Laser Therapy on Chemoradiotherapy-Induced Oral Mucositis and Salivary Inflammatory Mediators in Head and Neck Cancer Patients	Oton-Leite, AF. et al. (2015).	48 (7,5)	51 (8)	82 (13)	10.1002/lsm.22349	Randomized clinical study	Randomized clinical study evaluating the action of low-level laser therapy in the treatment of oral mucositis in patients with head and neck cancer undergoing chemotherapy and radiotherapy.
31	The use of low-energy laser (LEL) for the prevention of chemotherapy- and/or radiotherapy-induced oral mucositis in cancer patients: results from two prospective studies	Genot-Klastersky, M. et al. (2017)	45 (3,4)	-	80 (6,1)	10.1007/s00520-008-0439-8	Randomized clinical trial	Randomized clinical study evaluating low-level laser therapy as prevention of oral mucositis in head and neck cancer patients undergoing chemotherapy and radiotherapy.
32	Use of 660-nm Diode Laser in the Prevention and Treatment of Human Oral Mucositis Induced by Radiotherapy and Chemotherapy	Zanin, T. et al. (2010)	43 (3,9)	46 (4,1)	80 (7,1)	10.1089=pho.2008.2242	Non-randomized clinical study	Clinical study evaluating the preventive action of low-level laser therapy on oral mucositis in patients receiving chemotherapy and radiotherapy for the treatment of head and neck cancer.
33	Low-intensity red laser on the prevention and treatment of induced-oral mucositis in hamsters	Franca, CM. et al. (2009)	42 (3,4)	47 (3,9)	97 (8)	10.1016/j.jphotobiol.2008.09.006	In vitro experimental study	Experimental in vitro study that evaluating the action of low-level laser therapy in the prevention of chemically induced oral mucositis in hamsters.
34	Low-energy laser therapy for prevention of oral mucositis in hematopoietic stem cell transplantation	Jaguar, GC. et al. (2007)	39 (2,7)	46 (3,2)	81 (5,7)	10.1111/j.1601-0825.2006.01330.x	Non-randomized clinical study	Clinical study that evaluating laser therapy in the prevention of oral mucositis in hematopoietic cell transplant patients.
35	The use of low-level light therapy in supportive care for patients with breast cancer:	Robijns, J. et al. (2017)	39 (9,2)	42 (10,5)	63 (15,5)	10.1007/s10103-016-	Literature review	Literature review on the action of low-level laser therapy on oral mucositis in patients

	review of the literature					2056-y10		undergoing cancer treatment.
36	Effect of low-level laser therapy on inflammatory mediator release during chemotherapy-induced oral mucositis: a randomized preliminary study	Silva, GBL. et al. (2015)	38 (6,3)	41	74 (12)	10.1007/s10103-014-1624-2	Randomized clinical trial	Randomized clinical study that evaluating the action of low-level laser therapy in anti-inflammatory potential and the treatment of oral mucositis in chemotherapy patients.
37	Low-level laser for prevention and therapy of oral mucositis induced by chemotherapy or radiotherapy	Genot, MT. et al. (2005)	36 (2,2)	52 (3,2)	107 (6,6)	10.1097/01.cco.0000156196.22249.76	Literature review	Literature review on the action of low-level laser therapy in the prevention of oral mucositis in patients undergoing chemotherapy and radiotherapy.
38	Cost-effectiveness of the introduction of specialized oral care with laser therapy in hematopoietic stem cell transplantation	Bezinelli, LM. et al. (2014)	38 (5,1)	40 (5,5)	76 (9,5)	10.1002/hon.2050	Case-control	Retrospective case-control study evaluating the action of low-level laser therapy in the treatment of oral mucositis in hematopoietic cell transplant patients.
39	A systematic review and meta-analysis of the effect of low-level laser therapy (LLLT) on chemotherapy-induced oral mucositis in pediatric and young patients	He, MX. et al. (2018)	37 (11,6)	-	76 (22,6)	10.1007/s00431-017-3043-4	Systematic review with meta-analysis	Systematic review that evaluates the action of low-level laser therapy on oral mucositis in chemotherapy patients.
40	Effect of Class IV Laser Therapy on Chemotherapy-Induced Oral Mucositis A Clinical and Experimental Study	Ottaviani, G. et al. (2013)	36 (4,1)	39 (4,7)	71 (8,3)	10.1016/j.ajpath.2013.09.003	In vitro experimental study	Experimental in vitro study that evaluated the efficacy of low-frequency laser therapy in a model of oral mucositis in animals that obtained a chemotherapy regimen.
41	Low Level Helium Neon Laser therapy for chemoradiotherapy induced	Gautam, AP. et al. (2012)	33 (3,6)	32 (3,5)	70 (7,7)	10.1016/j.oraloncology.2012.03.008	Randomized clinical trial	Randomized clinical study evaluating the action of low-level laser therapy in the treatment of oral mucositis in patients with

	oral mucositis in oral cancer patients - A randomized controlled trial							head and neck cancer undergoing chemotherapy and radiotherapy.
42	Effect of intraoral low-level laser therapy on quality of life of patients with head and neck cancer undergoing radiotherapy	Oton-Leite, AF. et al. (2012)	32 (3,5)	35 (3,8)	56 (6,1)	10.1002/hed.21737	Randomized clinical trial	Randomized clinical study evaluating the action of low-level laser therapy in the treatment of oral mucositis in patients with head and neck cancer undergoing radiotherapy.
43	Low level laser therapy against radiation induced oral mucositis in elderly head and neck cancer patients-a randomized placebo controlled trial	Gautam, AP. et al. (2015)	30 (5)	33 (5,1)	66 (10,8)	10.1016/j.jphotobiol.2015.01.011	Randomized clinical trial	Randomized clinical study evaluating the action of low-level laser therapy in the prevention of oral mucositis in head and neck cancer patients receiving chemotherapy and radiotherapy.
44	Biomodulation of Inflammatory Cytokines Related to Oral Mucositis by Low-Level Laser Therapy	Basso, FG. et al. (2015)	30 (5)	30 (5)	54 (7,5)	10.1111/php.12445	In vitro experimental study	Experimental in vitro study evaluated the effects of low-level laser therapy on the expression of inflammatory cytokines related to the development of oral mucositis by gingival fibroblastos.
45	Low-level laser therapy for treatment of chemotherapy-induced oral mucositis in childhood: a randomized double-blind controlled study	Amadori, F. et al. (2016)	29 (5,8)	31 (6)	67 (9,8)	10.1007/s10103-016-1975-y	Randomized clinical trial	Randomized clinical study evaluating the action of low-level laser therapy in the treatment of oral mucositis in patients with head and neck cancer undergoing chemotherapy and radiotherapy.
46	Oral mucositis in pediatric patients undergoing hematopoietic stem cell transplantation: Clinical outcomes in a context of specialized oral care using	Eduardo, FD. et al. (2015)	29 (4,6)	30 (4,8)	53 (8,3)	10.1089/pho.2007.2225	Non-randomized clinical study	Clinical study evaluating the action of low-level laser therapy in the treatment of oral mucositis in hematopoietic cell transplant patients.

	low-level laser therapy							
47	Severity of Oral Mucositis in Patients Undergoing Hematopoietic Cell Transplantation and an Oral Laser Phototherapy Protocol: A Survey of 30 Patients	Eduardo, FD. et al. (2009)	28 (2,3)	31 (2,5)	69 (5,3)	10.1089/pho.2007.2225	Non-randomized clinical study	Clinical study evaluating the action of low-level laser therapy in the treatment of oral mucositis in hematopoietic cell transplant patients.
48	Cost-effectiveness of low-level laser therapy (LLLT) in head and neck cancer patients receiving concurrent chemoradiation	Antunes, HS. et al. (2016)	28 (5,4)	-	52 (9,4)	10.1016/j.oraloncology.2015.10.022	Randomized clinical trial	Randomized clinical study evaluating the action of low-level laser therapy in the treatment of oral mucositis in patients with head and neck cancer undergoing chemotherapy and radiotherapy.
49	Efficacy of low-level laser therapy as an auxiliary tool for management of acute side effects of head and neck radiotherapy	Gonzalez-Arriagada, WA. et al. (2018)	23 (7,3)	24 (8)	43 (13,3)	10.1080/14764172.2017.1376097	Case-control	Retrospective case-control study evaluating the efficacy of low-level laser therapy as an adjunct treatment of oral mucositis in head and neck cancer patients.
50	Low-level laser therapy prevents severe oral mucositis in patients submitted to hematopoietic stem cell transplantation: a randomized clinical trial	Ferreira, B. et al. (2016)	22 (4,4)	22 (9,6)	47 (4,2)	10.1007/s00520-015-2881-8	Randomized clinical trial	Randomized clinical study evaluating low-level laser therapy in the prevention of severe oral mucositis in patients undergoing hematopoietic cell transplantation.
